# The role of nanofat membranes in inhibiting bacterial growth: An in vitro analysis^☆^

**DOI:** 10.1016/j.jpra.2026.08.011

**Published:** 2026-08-19

**Authors:** Shahriar Nazari, Zahra Nejatipour, Mohammad Reza Pourani, Lessandro Martins, Paula Martin-Marfil, Saeedeh Ansari Dezfouli, Nabil Fakih-Gomez

**Affiliations:** a Department of ENT and Head and Neck Surgery, BMI Hospital, Tehran, Iran; b Private Practice. Zibaban Clinic, Management and Research of Complications Clinic, Tehran, Iran; c Department Breast Cancer Research Center (BCRC), Motamed Cancer Institute, Academic Center for Education, Culture and Research (ACECR), Tehran, Iran; d Skin Research Center, Shohada-e Tajrish Hospital, Shahid Beheshti University of Medical Sciences, Tehran, Iran; e Private Practice, 2653 Orion Business Health and Center, Goiania, Brazil; f Department of Endocrinology and Medical Nutrition, Salamanca University Hospital, Salamanca, Spain; g Clinical Laboratory of BMI, Tehran, Iran; h Department of Facial Plastic & Cranio-Maxillo-Facial Surgery, Fakih Hospital, Khaizaran, Lebanon; i Department of Surgery, University of Salamanca, Salamanca, Spain

**Keywords:** Antibacterial, Wound healing, Nanofat membrane, Platelet-rich fibrin, Regenerative medicine, Fat membrane

## Abstract

**Introduction:**

Wound healing is a major clinical concern, particularly in chronic wounds complicated by bacterial colonization, which delays healing and increases the risk of severe complications. Platelet-rich fibrin (PRF) and nanofat are promising regenerative biomaterials with potential antibacterial properties. This study evaluates the antibacterial efficacy of nanofat membranes, including nanofat-PRF and microfat-nanofat-PRF, against clinically relevant bacterial strains.

**Materials and methods:**

An experimental study was conducted using samples from five healthy volunteers. Nanofat membranes with varying ratios (nanofat:PRF 1:1, 1:2, 2:1, and microfat–nanofat–PRF 1:1:1) were prepared using autologous fat and injectable PRF. Antibacterial activity was tested against *Pseudomonas aeruginosa, Klebsiella pneumoniae, Staphylococcus aureus, E. coli*, and *Enterococcus faecalis* using the Kirby–Bauer disc diffusion method. Gentamicin and ampicillin served as positive controls, while injectable PRF and blank disks were used as negative controls. Data were analyzed using one-way and two-way ANOVA (p < 0.05).

**Results:**

The membranes exhibited substantial antibacterial activity against all tested strains, with *P. aeruginosa* showing the highest susceptibility. *S. aureus* revealed the lowest inhibition zone value in different fat membrane compositions. However, overall efficacy did not significantly differ between compositions. No inhibition was observed with injectable PRF alone.

**Conclusions:**

Nanofat membranes demonstrated notable antibacterial activity against major clinical pathogens, suggesting synergistic effects between adipose-derived and platelet components. Further studies with larger samples are warranted to identify the active antibacterial factors and optimize formulations for wound healing and infection control.

## Introduction

Wound healing is a major concern for clinicians and patients, particularly when acute wounds progress to chronic, non-healing lesions that present significant therapeutic challenges. Chronic wounds are often associated with persistent infections, prolonged pain, and extended hospital stays, leading to reduced quality of life and increased healthcare costs.^1^ Bacterial colonization further complicates healing by promoting inflammation and increasing the risk of severe complications, especially in patients with diabetes or surgical wounds.^2^ In diabetic foot ulcers, bacterial infection can delay healing, resulting in tissue necrosis and, in severe cases, amputation.^1^^,^^2^ The increasing prevalence of bacterial infections and antibiotic-resistant strains has intensified the search for alternative antimicrobial strategies, highlighting the need for innovative wound-healing technologies.^3^^,^^4^

Advances in tissue engineering have led to regenerative biomaterials, particularly autologous products such as platelet-rich plasma (PRP) and platelet-rich fibrin (PRF).^5^^,^^6^ PRF, a second-generation biomaterial, eliminates the need for coagulation while providing mechanical support and sustained growth factor release.^5^^,^^7^ Regenerative medicine increasingly utilizes PRF and nanofat for tissue repair, with both demonstrating potential antibacterial properties.^8^ However, their liquid form presents clinical limitations, leading to the development of the nanofat-PRF membrane, a third-generation autologous material with improved stability.^8^ Nanofat, rich in adipose-derived stem cells (ADSCs), promotes collagen production, while PRF provides a fibrin scaffold that supports sustained tissue repair.^7^^,^^9^, ^10^, ^11^, ^12^, ^13^, ^14^

The nanofat-PRF membrane (known in the literature as nanofat membrane) demonstrated healing benefits in the treatment of chronic wounds, diabetic ulcers, and cosmetic and reconstructive procedures.^8^ Additionally, it offers practical advantages, such as ease of handling, the ability to cover extensive wound areas, and the option for secure fixation with sutures.^8^ Furthermore, this membrane showed significant vascularization, with endothelial markers indicating potential for vascular neoformation.^15^ Histopathological analysis revealed adipose tissue fragmentation, vascular proliferation, and platelet/fibrin deposition, suggesting tissue remodeling.^15^

Combining these biomaterials may enhance efficacy against a broader spectrum of pathogens due to their individual antimicrobial properties. This study aimed to evaluate the antibacterial activity of nanofat-PRF and microfat-nanofat-PRF membranes against clinically relevant bacterial strains, including *Pseudomonas aeruginosa, Klebsiella pneumoniae, Staphylococcus aureus, E. coli,* and *Enterococcus faecalis*. Additionally, we assessed the effectiveness of different nanofat membrane compositions in inhibiting bacterial growth and the differential susceptibility of these species, providing insight into their potential as a novel approach for infection control.

## Material and methods

An experimental study was conducted in July 2025 and August 2025 and included five healthy volunteers who attended our clinic during that period. All participants were thoroughly informed about the study design and objectives and provided written informed consent prior to enrollment. The inclusion criteria comprised healthy, non-obese, non-diabetic adults aged 20 to 55 years, free from systemic or dermatological diseases, and with no history of antibiotic, anti-inflammatory, or immunosuppressive medication use within the preceding three months. Exclusion criteria included a history of infectious, autoimmune, or hematologic disorders; pregnancy or breastfeeding; current use of anticoagulant or antiplatelet therapy; and inability or unwillingness to provide informed consent.

### Nanofat membranes preparation^8^

The procedure begins with fat harvesting through a small 2 mm incision in the donor area (Lower abdomen). A modified Klein's tumescent solution (500 mL normal saline, 10 mL 2% lidocaine, and 1 mL of 1:1000 adrenaline) is infiltrated slowly using a tumescent infiltrator. Approximately 10 ml of subcutaneous fat is manually harvested using a Tonnard harvester, a 2.4 mm x 20 cm cannula with 1 mm diameter sharp holes, connected to a 10-ml Luer-Lock syringe. The harvested syringe is capped in a vertical position to allow separation of layers over 4–6 min. The yellow adipose grafts (microfat) separate by density from the infranatant fluid, forming a lipid layer on top. The tumescent solution is drained, and the microfat is transferred to 10 mL syringes, excluding the oil layer. A single wash with Ringer's lactate solution reduces residual local anesthetic and red blood cells. The microfat is decanted a second time to complete preparation.

Emulsification involves three transfers, beginning with cleaned microfat loaded into 20 mL syringes. The fat is mechanically emulsified by shifting the contents 20 times back and forth between two connected 20 mL syringes using a 2.4 mm connector. This is repeated 20 times each with a 1.4 mm and a 1.2 mm connector, liquefying the fat to a pale-yellow appearance. The emulsified fat is then passed once through a nano-transfer block with a double filter (600 µm and 400 µm), resulting in nanofat.

For injectable PRF preparation, 9 ml of venous blood is drawn from the patient using an 18 G needle, collected in sterile 9 ml plain tubes without anticoagulant. Blood is divided equally into tubes, placed symmetrically around the rotor axis of a fixed-angle centrifuge (HwLAB, Model HW12M) for balance. The centrifuge operates at 4000 rpm (G-force = 1340) for 1 min at room temperature (22 °C), generating approximately 3.5 mL of injectable PRF.^8^

To create the nanofat membrane, the Fat Membrane Device is prepared to the desired length by setting a vertical stopper for membrane sizes of 1 cm x 1 cm. Within 1 min of plasma preparation, the injectable PRF is transferred into the device before it gelifies. Nanofat is then blended with the plasma using a sterile rod. As the plasma matures into a solid PRF, incorporating the nanofat, it develops a thick, gelatinous consistency, acquiring a yellowish hue. The membrane typically sets within 5–10 min. After setting, the vertical stopper is removed, and the device's lid gently compresses the fat-fibrin gel to form a uniform membrane. This compression extracts excess liquid and converts the gel into a dense fibrin membrane without slicing. All membranes were prepared using the same previously published standardized technique.^8^ The thickened nanofat-PRF membrane is then carefully removed and divided into small pieces for further analysis.

### Bacterial strain preparation and culture conditions

The antibacterial efficacy of nanofat membranes was evaluated against five bacterial strains obtained from the American Type Culture Collection (ATCC). Mueller-Hinton Broth (Sigma Aldrich, 70,192) was inoculated with the following pathogens: *Pseudomonas aeruginosa* (ATCC 27,853), *Klebsiella pneumoniae* (ATCC 13,883), *Staphylococcus aureus* (ATCC 25,923), *E. coli* (ATCC 25,922), and *Enterococcus faecalis* (ATCC 29,212). After incubation at 37 °C, bacterial cultures were standardized to a concentration of 0.5 McFarland (1.5 × 10^8 CFU/mL). Mueller-Hinton Agar (Sigma Aldrich, 70,191) plates were inoculated with 200 µL of each bacterial suspension using a sterile swab and incubated at 37 °C for 16 h to form a continuous culture. Additional agar plates were prepared for the five bacteria*,* resulting in two plates for each of these strains.

The bacterial strains used in this study were selected because they are among the most common pathogens involved in surgical site infections, chronic wounds, and hospital-acquired infections. They include both Gram-positive (*S. aureus, E. faecalis*) and Gram-negative (*P. aeruginosa, K. pneumoniae, E. coli*) bacteria, providing a broad representation of clinically relevant microorganisms with diverse structural and resistance characteristics. These pathogens are frequently isolated from infected wounds and postoperative infections, particularly in hospitalized or immunocompromised patients. Therefore, their inclusion allowed evaluation of the antibacterial activity of nanofat membranes against a representative spectrum of clinically relevant bacteria.

### Experimental setup

**Fig. 1:**
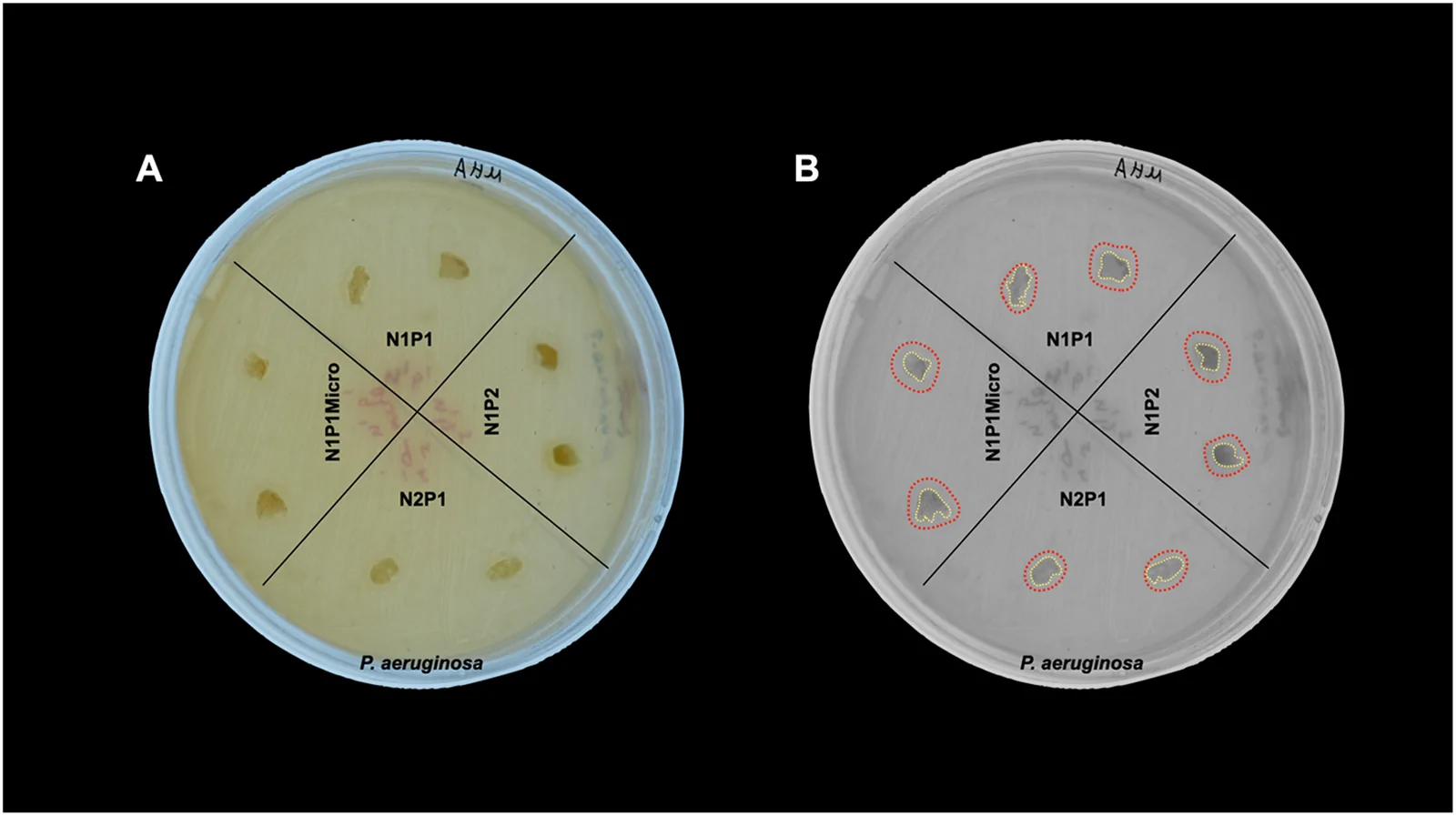
Antibacterial activity of nanofat–PRF membranes against *P. aeruginosa* were evaluated using the Kirby–Bauer disc diffusion method. Image A shows the same field as image B, presented in black and white for contrast enhancement. Four types of membranes (outlined by yellow dotted lines in image B) were tested: N1P1, N2P1, N1P2, and N1P1Micro, corresponding to nanofat:PRF ratios of 1:1, 2:1, 1:2, and a 1:1:1 microfat–nanofat–PRF composition, respectively. Within each quadrant, two membranes of the same type were placed to allow precise measurement of the mean radius of the inhibition zones. The inhibition zone radius (indicated by the red dotted line in image B) was measured in millimeters using a caliper. Note: N is nanofat, P is PRF and Micro is mircrofat.

**Fig. 2:**
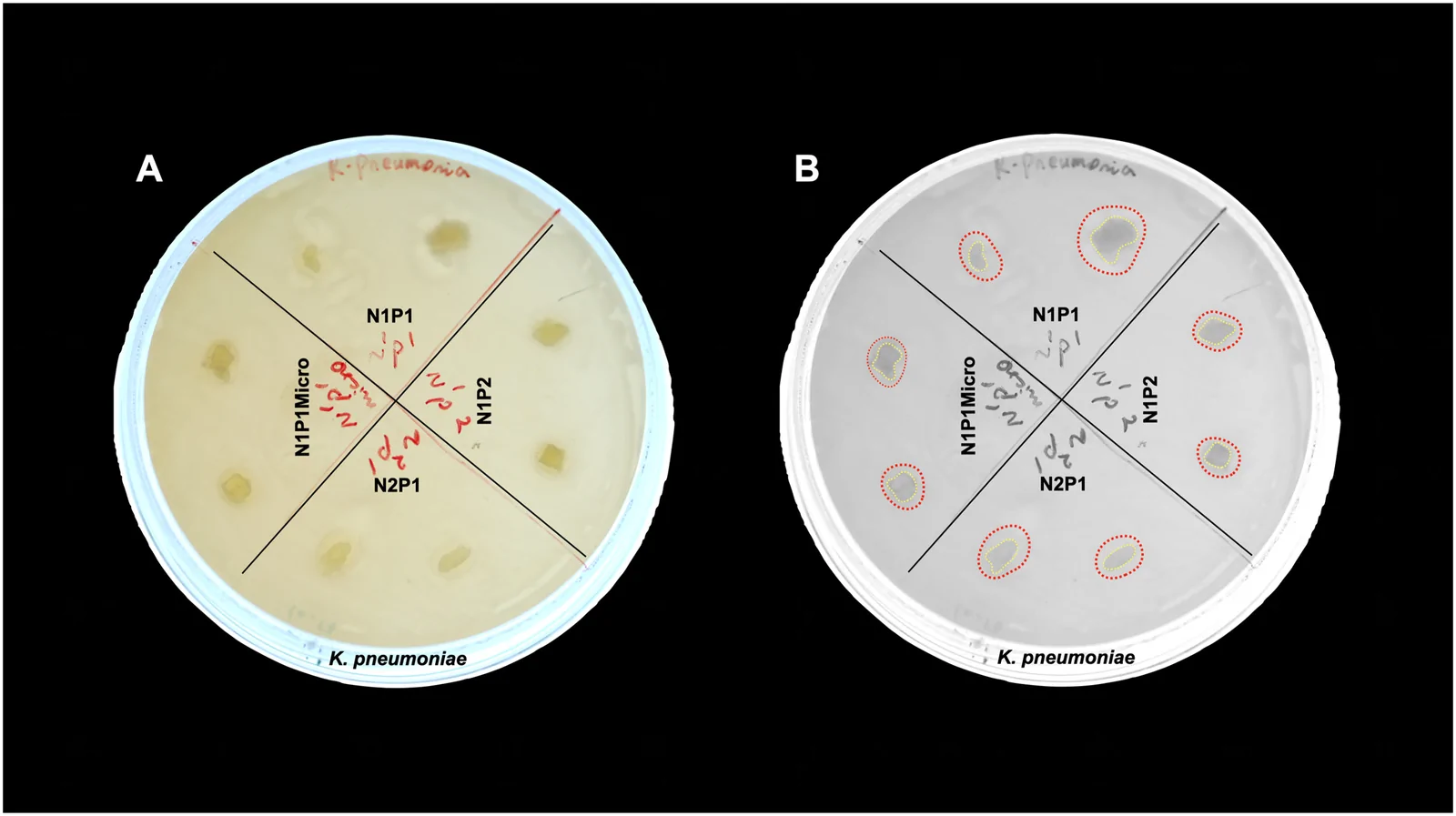
Antibacterial activity of nanofat–PRF membranes against *K. pneumoniae* were evaluated using the Kirby–Bauer disc diffusion method. Image A shows the same field as image B, presented in black and white for contrast enhancement. Four types of membranes (outlined by yellow dotted lines in image B) were tested: N1P1, N2P1, N1P2, and N1P1Micro, corresponding to nanofat:PRF ratios of 1:1, 2:1, 1:2, and a 1:1:1 microfat–nanofat–PRF composition, respectively. Within each quadrant, two membranes of the same type were placed to allow precise measurement of the mean radius of the inhibition zones. The inhibition zone radius (indicated by the red dotted line in image B) was measured in millimeters using a caliper. Note: N is nanofat, P is PRF and Micro is mircrofat.

**Fig. 3:**
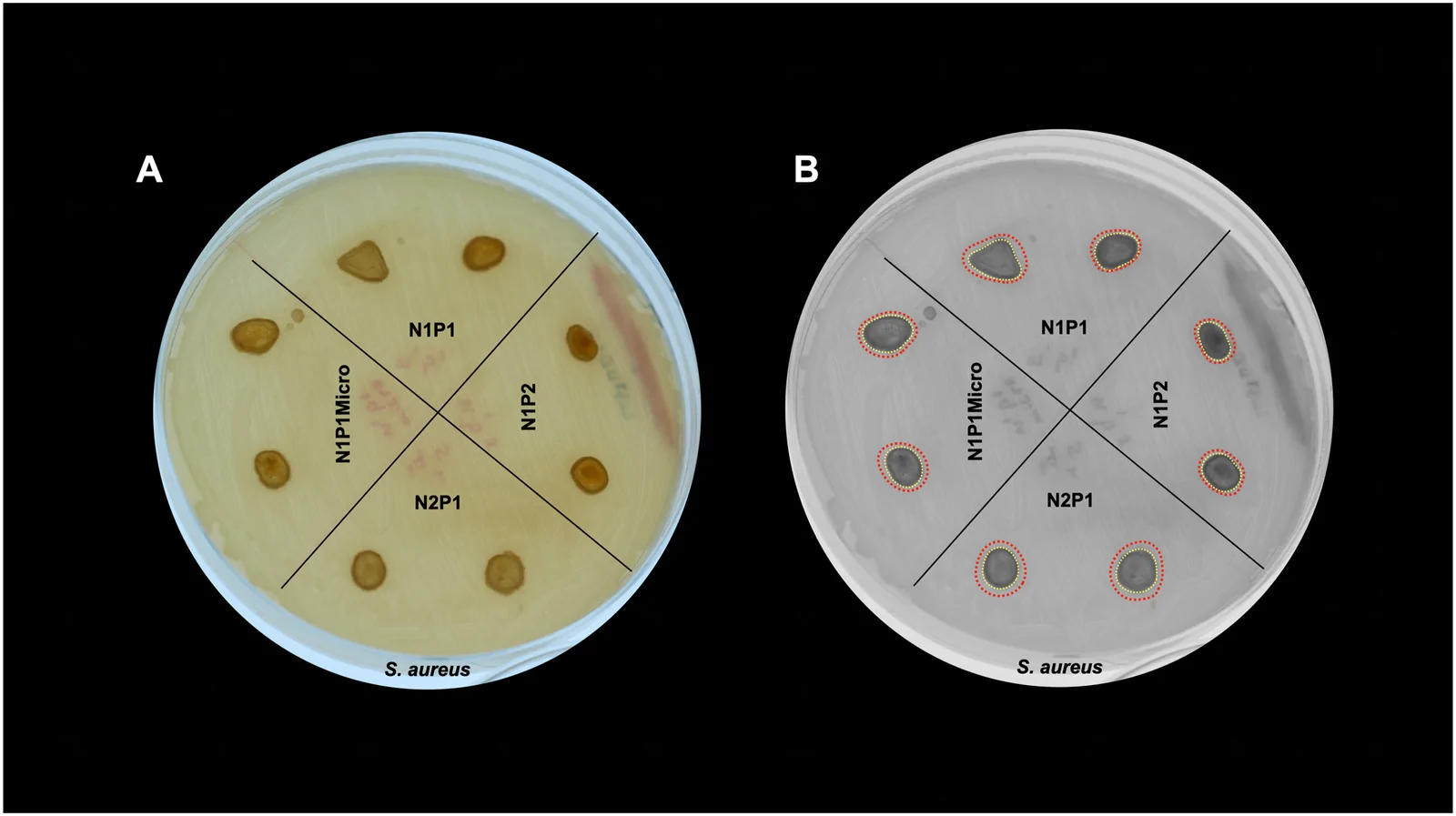
Antibacterial activity of nanofat–PRF membranes against *S. aureus* were evaluated using the Kirby–Bauer disc diffusion method. Image A shows the same field as image B, presented in black and white for contrast enhancement. Four types of membranes (outlined by yellow dotted lines in image B) were tested: N1P1, N2P1, N1P2, and N1P1Micro, corresponding to nanofat:PRF ratios of 1:1, 2:1, 1:2, and a 1:1:1 microfat–nanofat–PRF composition, respectively. Within each quadrant, two membranes of the same type were placed to allow precise measurement of the mean radius of the inhibition zones. The inhibition zone radius (indicated by the red dotted line in image B) was measured in millimeters using a caliper. Note: N is nanofat, P is PRF and Micro is mircrofat.

**Fig. 4:**
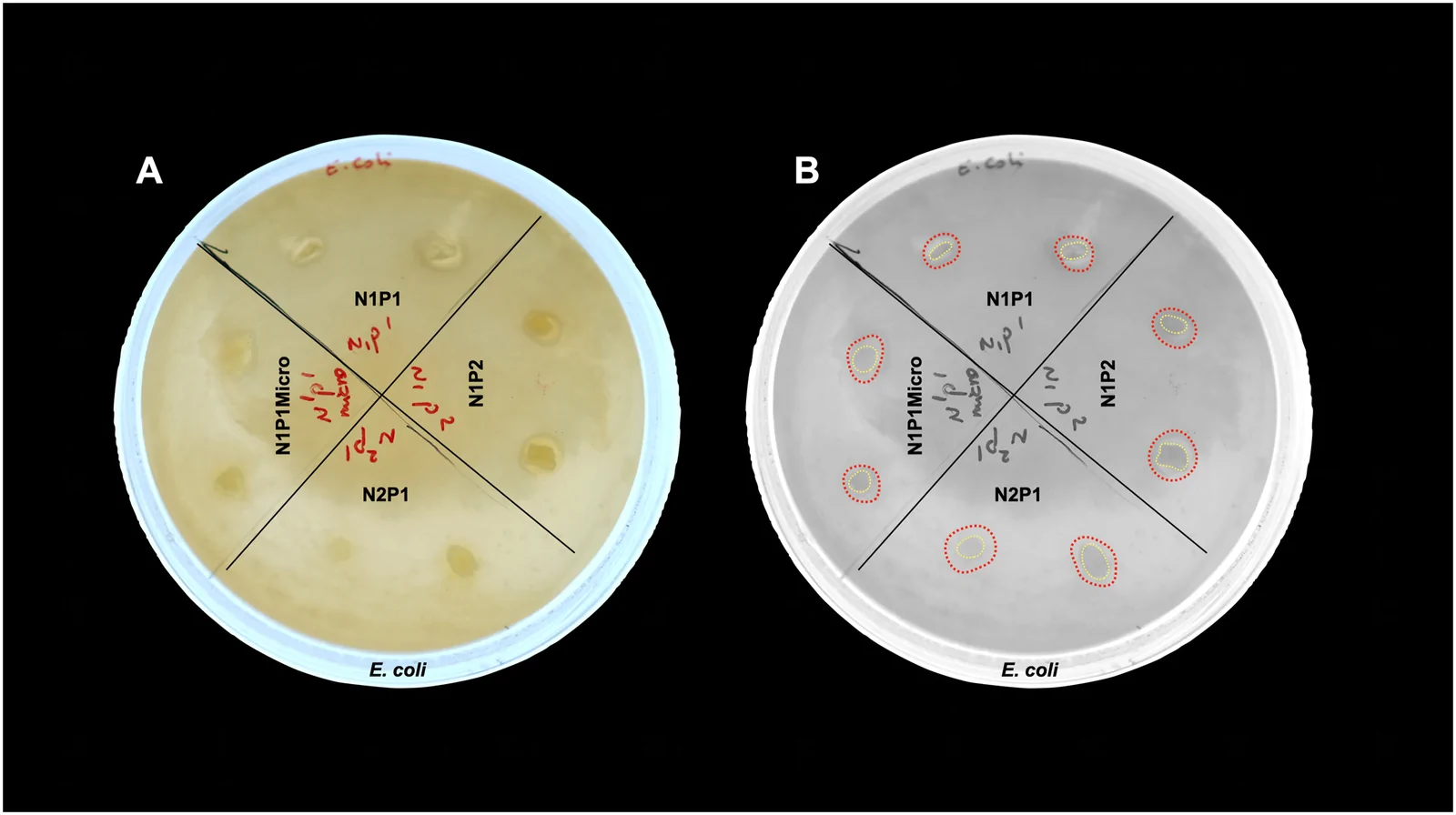
Antibacterial activity of nanofat–PRF membranes against *E. coli* were evaluated using the Kirby–Bauer disc diffusion method. Image A shows the same field as image B, presented in black and white for contrast enhancement. Four types of membranes (outlined by yellow dotted lines in image B) were tested: N1P1, N2P1, N1P2, and N1P1Micro, corresponding to nanofat:PRF ratios of 1:1, 2:1, 1:2, and a 1:1:1 microfat–nanofat–PRF composition, respectively. Within each quadrant, two membranes of the same type were placed to allow precise measurement of the mean radius of the inhibition zones. The inhibition zone radius (indicated by the red dotted line in image B) was measured in millimeters using a caliper. Note: N is nanofat, P is PRF and Micro is mircrofat.

**Fig. 5:**
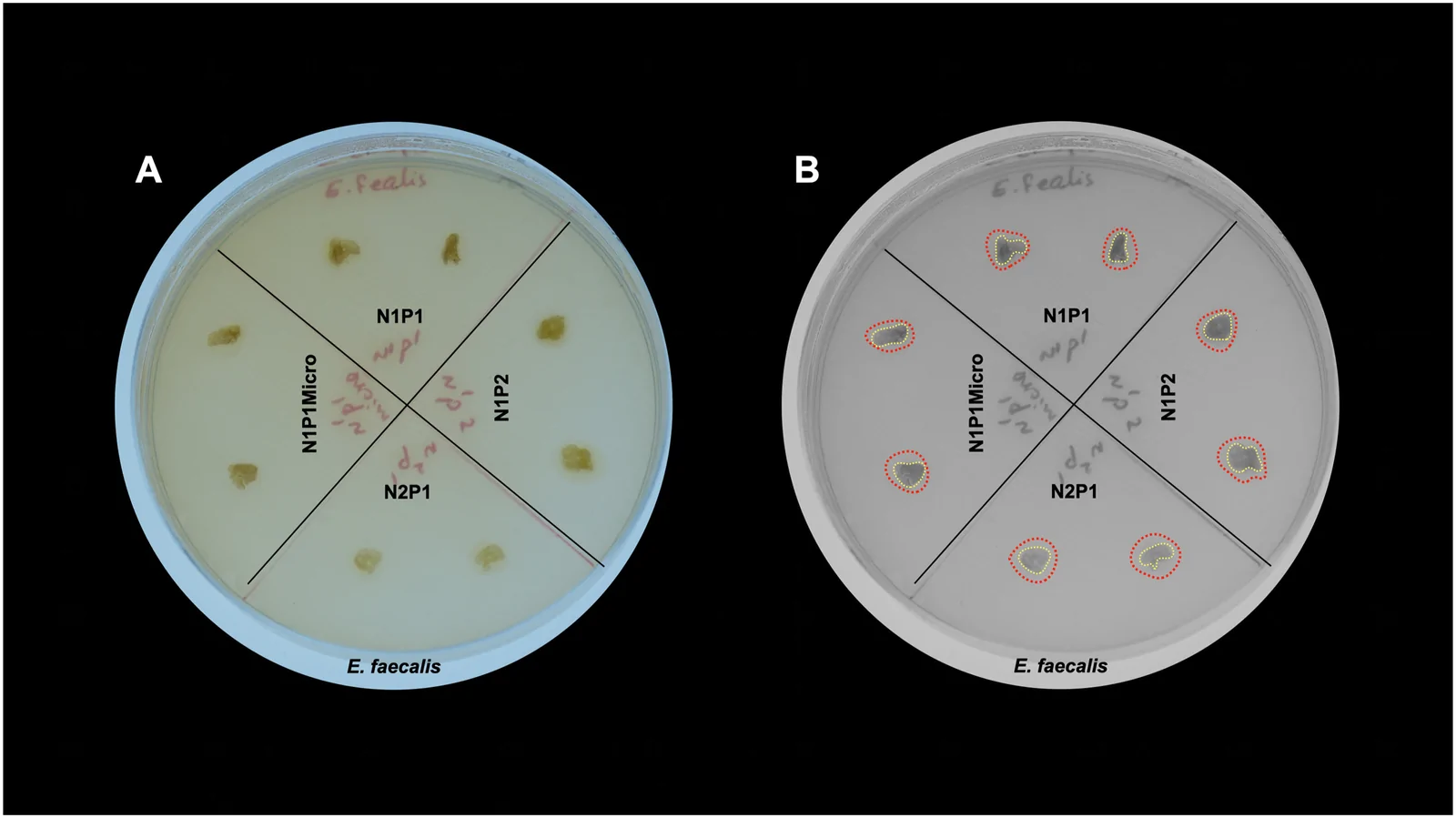
Antibacterial activity of nanofat–PRF membranes against *E. faecalis* were evaluated using the Kirby–Bauer disc diffusion method. Image A shows the same field as image B, presented in black and white for contrast enhancement. Four types of membranes (outlined by yellow dotted lines in image B) were tested: N1P1, N2P1, N1P2, and N1P1Micro, corresponding to nanofat:PRF ratios of 1:1, 2:1, 1:2, and a 1:1:1 microfat–nanofat–PRF composition, respectively. Within each quadrant, two membranes of the same type were placed to allow precise measurement of the mean radius of the inhibition zones. The inhibition zone radius (indicated by the red dotted line in image B) was measured in millimeters using a caliper. Note: N is nanofat, P is PRF and Micro is mircrofat.

Three nanofat–PRF membrane compositions were prepared with nanofat-to-PRF ratios of 1:1, 1:2, and 2:1, along with an additional composition featuring a 1:1:1 microfat–nanofat–PRF ratio. All membranes were disc-shaped, with dimensions of approximately 6 mm × 6 mm (±1 mm). These compositions were derived from samples obtained from five patients, including healthy individuals with no history of antibiotic consumption for the past three months (Fig. 1, Fig. 2, Fig. 3, Fig. 4, Fig. 5). Fig. 4, Fig. 3, Fig. 2

The membranes were placed on inoculated agar surfaces according to the Kirby–Bauer disc diffusion method. Experiments were performed on blood agar plates (Sigma-Aldrich, 70,133) to improve visualization of inhibition zones against the agar background. All experiments were conducted in duplicate for each patient sample and bacterial strain. Each plate was divided into four quadrants, with each quadrant containing one of the four membrane compositions. Within each quadrant, two membranes of the same type were placed to enable accurate determination of inhibition zones (Fig. 1, Fig. 2, Fig. 3, Fig. 4, Fig. 5). Antibacterial activity was quantified by measuring the radius of the inhibition zone from the membrane edge to the boundary of visible bacterial growth inhibition using a digital caliper with a precision of 0.01 mm (Harley Benton). For each membrane, the maximum and minimum radii were recorded and averaged to reduce measurement variability.

**Fig. 6:**
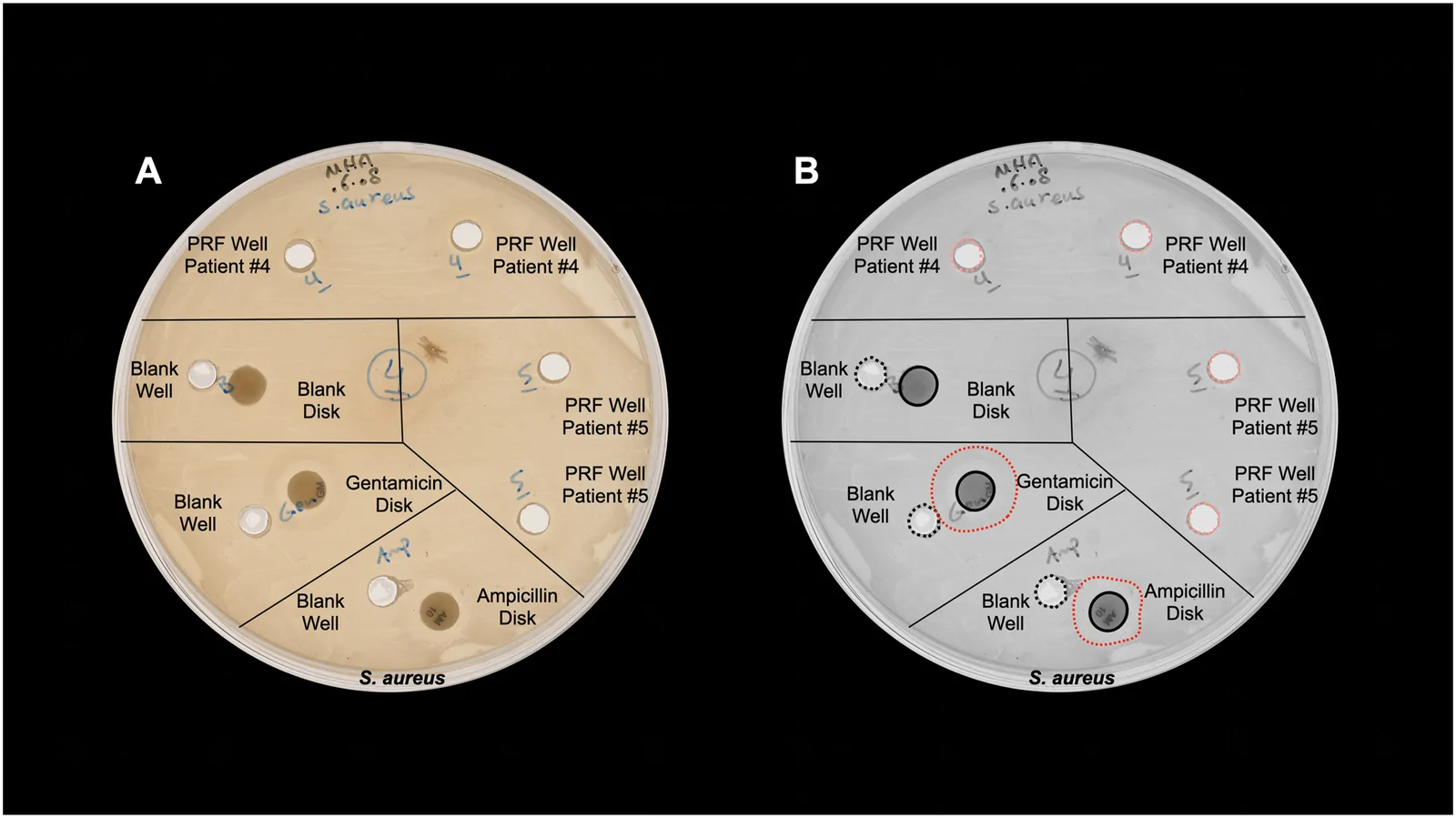
The antibacterial activity of injectable PRF against *S. aureus* was evaluated using the well disc diffusion method. Image A depicts the same field as image B, shown in black and white to enhance contrast. PRF samples (outlined by pink dotted lines in image B), blank wells (outlined by black dotted lines), a gentamicin disc (outlined by a black circle and labeled Gentamicin disc), and an ampicillin disc (outlined by a black circle and labeled Ampicillin disc) were tested using the well-diffusion method. For each patient (Patient 4 and Patient 5), two PRF samples were placed to determine the mean radius of the resulting inhibition zones, along with one blank well adjacent to a blank disc (control), one well with a gentamicin disc adjacent to it, and one well with an ampicillin disc adjacent to it. The inhibition zone radii (indicated by the red dotted line) were measured in millimeters using a caliper. Note: PRF = platelet-rich fibrin.

**Fig. 7:**
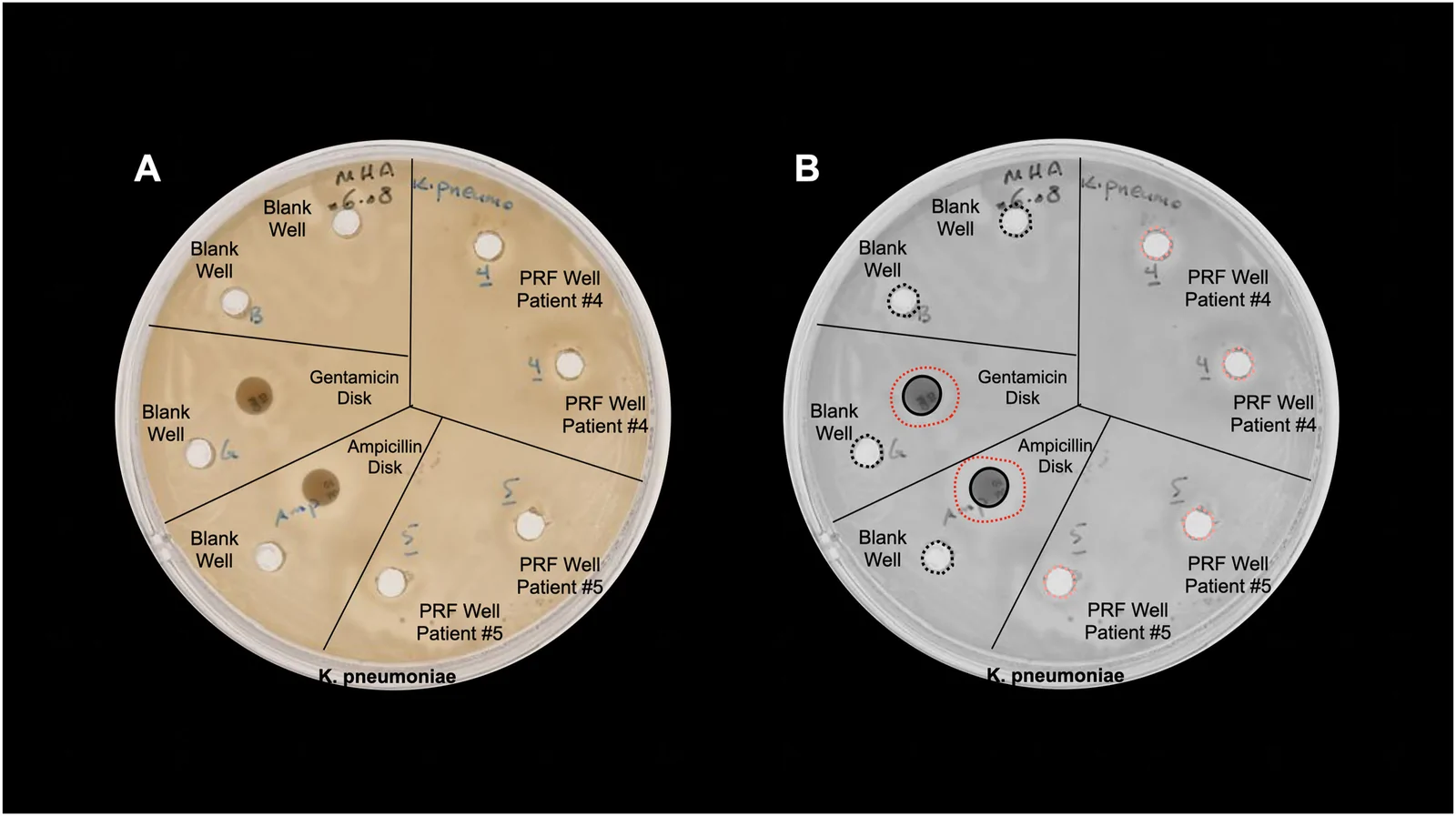
The antibacterial activity of injectable PRF against *K. pneumoniae* was evaluated using the well disc diffusion method. Image A depicts the same field as image B, shown in black and white to enhance contrast. PRF samples (outlined by pink dotted lines in image B), blank wells (outlined by black dotted lines), a gentamicin disc (outlined by a black circle and labeled Gentamicin disc), and an ampicillin disc (outlined by a black circle and labeled Ampicillin disc) were tested using the well-diffusion method. For each patient (Patient 4 and Patient 5), two PRF samples were placed to determine the mean radius of the resulting inhibition zones, along with two blank well (control), one well with a gentamicin disc adjacent to it, and one well with an ampicillin disc adjacent to it. The inhibition zone radii (indicated by the red dotted line) were measured in millimeters using a caliper. Note: PRF = platelet-rich fibrin.

The antibacterial activity of injectable PRF samples collected from five patients was evaluated using the well-diffusion method to establish a baseline for comparison with nanofat-PRF compositions (Fig. 6, Fig. 7). Gentamicin and ampicillin disks served as positive controls, while blank disks and empty agar (blank) wells were included as negative controls under identical experimental conditions (Fig. 6, Fig. 7).

### Statistical analysis

The inhibition zone measurements obtained from replicated experiments were used for statistical comparisons. Data were analyzed using one-way and two-way ANOVA with SPSS 26 software. A p-value < 0.05 was considered statistically significant.

## Results

**Fig. 8:**
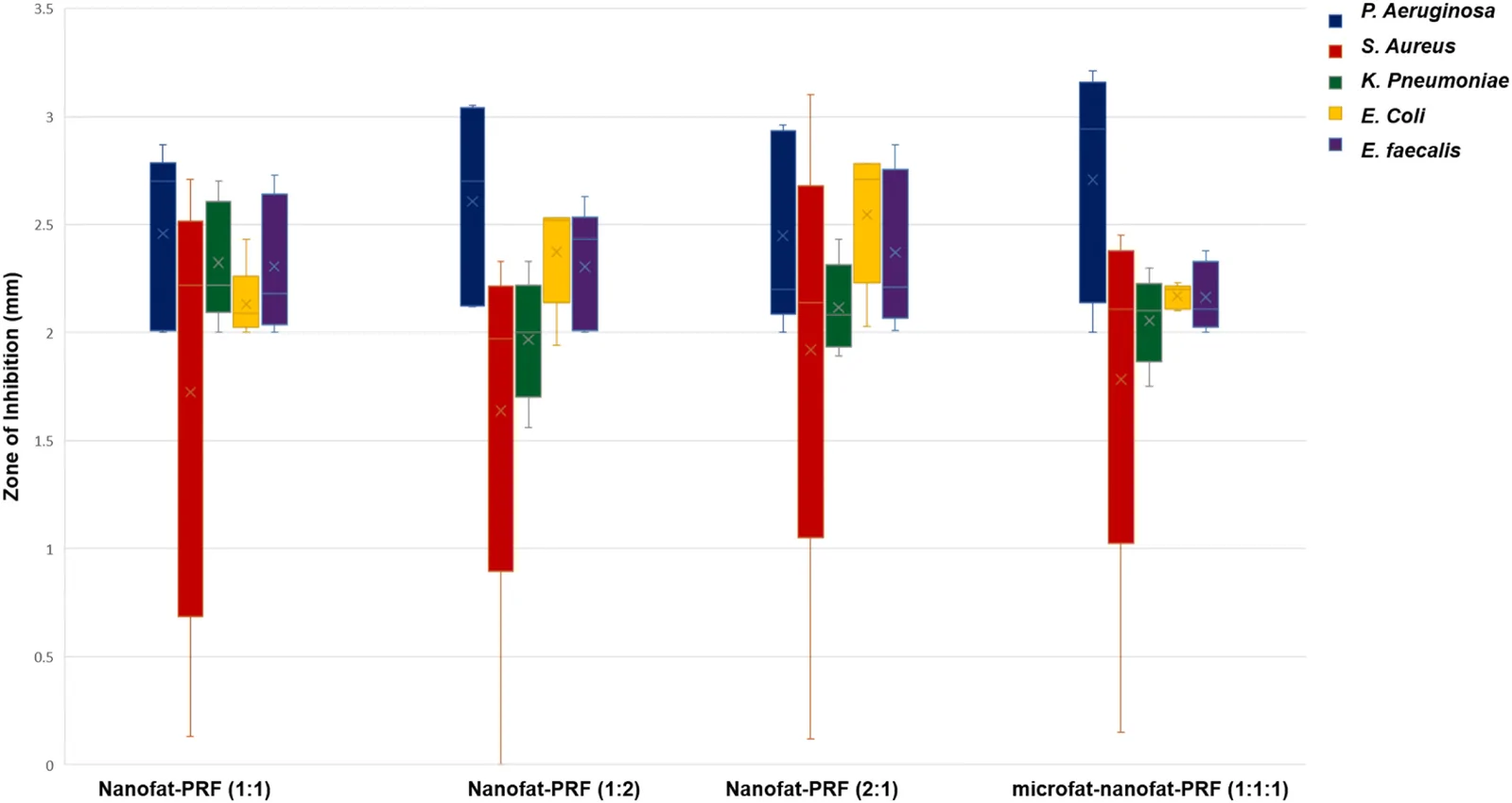
The zone of inhibition in different types of fat membranes (1:1, 1:2, and 2:1 nanofat-to-PRF ratios, and a 1:1:1 microfat-nanofat-PRF ratio) across different bacterial strains.

Statistical analysis demonstrated that all nanofat membranes (with or without microfat) in combination with injectable PRF exhibited antibacterial activity, inhibiting the growth of pathogens, including *P. aeruginosa, K. pneumoniae, S. aureus, E. coli*, and *E. faecalis*. The zone of inhibition for *P. aeruginosa, K. pneumoniae, S. aureus, E. coli*, and *E. faecalis* was 2.55 ± 0.44 mm, 2.12 ± 0.26 mm, 1.76 ± 0.93 mm, 2.30 ± 0.27 mm, and 2.29 ± 0.28 mm, respectively. Any inhibition zone greater than 0 mm was considered indicative of antibacterial activity. Complete data on the zone of inhibition are presented in Table 1 and Fig. 8. The maximum zone of inhibition for *S. aureus, E. coli*, and *E. faecalis* was observed with a nanofat:PRF ratio of 2:1 (P > 0.05), while the maximum susceptibility for *P. aeruginosa* and *K. pneumoniae* occurred with a microfat-nanofat-PRF and nanofat:PRF ratio of 1:1, respectively (P > 0.05), which was all insignificant. *S. aureus* exhibited the lowest zone of inhibition, ranging from 0 to 0.15 mm across different fat membrane compositions, suggesting the least effect of fat membranes on this bacterium. Moreover, the maximum zone of inhibition in total was detected in a nanofat:PRF ratio of 2:1.

**Table 1:** Data on the zone of inhibition of all nanofat membranes (with or without microfat) in combination with PRF for *P. aeruginosa, K. pneumoniae, S. aureus, E. coli*, and *E. faecalis*.

Bacteria Type	Fat Membrane Type	Number of Samples	Zone of Inhibition (Mean)	Standard Deviation
*Pseudomonas aeruginosa*	Nanofat:PRF (1:1)	5	2.458	.414
	Nanofat:PRF (1:2)	5	2.606	.460
	Nanofat:PRF (2:1)	5	2.448	.451
	Microfat:Nanofat:PRF (1:1:1)	5	2.708	.536
	Total	20	2.555	.443
*Klebsiella pneumoniae*	Nanofat:PRF (1:1)	5	2.324	.278
	Nanofat:PRF (1:2)	5	1.968	.289
	Nanofat:PRF (2:1)	5	2.116	.210
	Microfat:Nanofat:PRF (1:1:1)	5	2.056	.205
	Total	20	2.116	.265
*Staphylococcus aureus*	Nanofat:PRF (1:1)	5	1.724	1.042
	Nanofat:PRF (1:2)	5	1.638	.936
	Nanofat:PRF (2:1)	5	1.920	1.095
	Microfat:Nanofat:PRF (1:1:1)	5	1.784	.936
	Total	20	1.766	.928
*E. coli*	Nanofat:PRF (1:1)	5	2.132	.170
	Nanofat:PRF (1:2)	5	2.372	.254
	Nanofat:PRF (2:1)	5	2.546	.322
	Microfat:Nanofat:PRF (1:1:1)	5	2.170	.056
	Total	20	2.305	.267
*Enterococcus faecalis*	Nanofat:PRF (1:1)	5	2.306	.318
	Nanofat:PRF (1:2)	5	2.304	.280
	Nanofat:PRF (2:1)	5	2.370	.367
	Microfat:Nanofat:PRF (1:1:1)	5	2.164	.160
	Total	20	2.286	.278
Total	Nanofat:PRF (1:1)	25	2.188	.558
	Nanofat:PRF (1:2)	25	2.177	.581
	Nanofat:PRF (2:1)	25	2.280	.579
	Microfat:Nanofat:PRF (1:1:1)	25	2.176	.547
	Total	100	2.205	.560

The antimicrobial effects of the different fat membranes were generally similar across bacteria, with the exception of *E. coli*. The nanofat:PRF ratio of 2:1 was more effective than the 1:1 ratio (zone of inhibition: 2.55 mm vs. 2.13 mm, respectively; P = 0.045). Overall, no significant differences were observed in the zone of inhibition among the different fat membrane types across all bacteria (P = 0.9).

To evaluate the potential antibacterial effects of injectable PRF (authors protocol), the inhibition zones of injectable PRF samples were measured. The inhibition zones for all five bacterial strains were 0 mm, as were those observed in the blank disks and empty agar wells (Fig. 6, Fig. 7). In contrast, the mean diameters of the inhibition zones produced by gentamicin disks were 2.35, 3.15, 3.15, 6.70, and 8.15 mm, and those produced by ampicillin disks were 4.65, 4.30, 4.10, 6.55, and 5.90 mm for *P. aeruginosa, S. aureus, K. pneumoniae, E. faecalis, and E. coli*, respectively.

## Discussion

Bacterial colonization delays healing, particularly in vulnerable patients such as those with diabetes, increasing the risk of severe complications. The rise of antibiotic-resistant strains further highlights the need for innovative wound-healing strategies.^2^^,^^4^

Advances in tissue engineering have led to the development of regenerative biomaterials, particularly autologous sources such as PRP and PRF.^5^^,^^6^ PRP promotes healing through the release of growth factors but initially required a coagulating agent, limiting its clinical practicality.^16^, ^17^, ^18^ PRP has also been shown to inhibit biofilm formation and reduce bacterial growth, suggesting potential benefits in infection management.^19^^,^^20^ PRF was introduced as a second-generation biomaterial that eliminates the need for coagulation while providing mechanical support and sustained growth factor release.^5^^,^^7^ This simplified preparation has facilitated its widespread use in plastic surgery, dentistry, and regenerative medicine.^21^^,^^22^ A systematic review by Moraschini et al. reported that PRF demonstrates antimicrobial activity against several pathogens, particularly bacteria.^23^ Platelets also express toll-like receptors (TLRs), which trigger inflammatory signaling pathways and the release of cytokines that modulate leukocyte responses during infection and inflammation.^24^

Nanofat, rich in mesenchymal stem cells, growth factors, and cytokines, has been widely reported to support tissue repair and regeneration. Clinical evidence that adipose-derived grafts are biologically active beyond simple volumization was demonstrated by Rigotti et al.,^25^ who reported symptomatic improvement and tissue regeneration with neovascularization following autologous lipoaspirate transplantation for severe radiotherapy-induced tissue injury, together with the identification of mesenchymal stem/stromal cell–like populations within the stromal vascular fraction. Experimental evidence further suggests that mesenchymal stem/stromal cells may contribute directly to antibacterial defense. Krasnodembskaya et al. demonstrated that human mesenchymal stem cells exhibit antimicrobial activity partly through secretion of the antimicrobial peptide LL-37, which disrupts bacterial membranes and contributes to innate immune defense.^26^ Although these mechanisms were not directly investigated in the present study, they provide a plausible biological explanation for the antibacterial effects observed in nanofat membranes.

Sanchez-Macedo et al. also reported that nanofat treatment upregulates inflammatory, antimicrobial, and wound-healing pathways, supporting its potential role in infection control.^27^ During bacterial infection, adipocyte precursors can differentiate into adipocytes capable of releasing antimicrobial peptides such as cathelicidin, which may contribute to antibacterial defense.^28^

Regenerative medicine increasingly employs autologous materials such as PRF and nanofat for tissue repair.^8^ Surgical site infections (SSIs) can delay healing and worsen clinical outcomes, and both nanofat and PRF have attracted interest for their potential antibacterial properties.^27^^,^^29^ When combined in liquid form, these biomaterials have shown benefits in reducing infection-related complications in reconstructive surgery.^30^^,^^31^ However, their liquid form presents handling limitations, leading to the development of the nanofat-PRF membrane, a third-generation autologous material with improved stability and regenerative potential.^8^ Nanofat promotes collagen production and tissue regeneration through ADSCs,^11^^,^^14^ while PRF provides a fibrin scaffold for sustained growth factor release.^12^^,^^13^ Fakih-Gomez et al. demonstrated that the nanofat membrane shows promise as a regenerative scaffold, with significant vascularization evidenced by endothelial markers such as CD31, CD34, and ERG.^15^ Histopathological analysis also revealed fragmented adipose tissue, vascular proliferation, platelet accumulation, and fibrin deposition, suggesting active tissue remodeling.^15^

Given their individual antimicrobial properties, combining these biomaterials could enhance their efficacy against a broader spectrum of pathogens. The combination of leukocyte-rich PRP and nanofat has been shown to be effective in the treatment of nonhealing infected wounds, as PRP enhances both the regenerative and antibacterial efficacy of fat grafting.^32^

This study confirms that all nanofat membranes exhibit antibacterial activity. However, the significant variation in bacterial strain response underscores the differential susceptibility of various bacterial species to the antibacterial effects of nanofat membranes. *P. aeruginosa* exhibited greater susceptibility compared to the other strains, suggesting that the active compounds in the nanofat membranes may target specific mechanisms in this bacterium. However, the authors did not observe any antibacterial activity using their injectable PRF (iPRF) protocol (plain tubes containing whole blood centrifuged for 1 min at 4000 rpm, corresponding to a relative centrifugal G-force of 1340), suggesting that its combination with fat may exert a synergistic antibacterial effect. In contrast, several studies have highlighted the antimicrobial properties of iPRF, which has also been employed as a local drug delivery vehicle for certain antibiotics.^30^, ^31^, ^32^, ^33^ The type of iPRF used in our study was selected for its uniform plasma composition, which provides optimal aesthetic and regenerative characteristics. Notably, the applied G-force and the type of centrifuge rotor can substantially influence leukocyte concentration and its associated antimicrobial activity. We emphasize the importance of activating innate immunity, particularly macrophage activation, following the application of the nanofat membrane, which may result from the co-activation of iPRF and nanofat. While the present findings demonstrate measurable antibacterial activity of nanofat membranes in vitro, the biological mechanisms responsible for this effect were not directly investigated in this study and therefore remain speculative.

The notably smaller zone of inhibition in *S. aureus* compared to other strains may indicate a weaker response of *S. aureus* to the nanofat membranes. These findings align with the understanding that, as Gram-positive bacteria, staphylococci may exhibit different susceptibility profiles than Gram-negative bacteria, possibly due to differences in cell membrane structures and other resistance mechanisms.

The findings suggest that nanofat membranes could serve as effective adjuncts in treating bacterial infections, particularly those caused by *P. aeruginosa*, which demonstrated higher susceptibility. The significant increased sensitivity of *E. coli* to the 2:1 nanofat:PRF composition indicates that this formulation may show notable activity against *E. coli*, as well as other bacteria such as *S. aureus* and *E. faecalis*.

The differences in the cell wall structures of Gram-positive and Gram-negative bacteria may explain this differential susceptibility. The absence of overall significance between different fat compositions and bacterial strains may reflect the complexity of interactions between these variables.

The findings of this study are preliminary due to the limited sample size, highlighting the need for future investigations with larger cohorts and appropriate power analyses to validate and generalize these results. The small sample population may limit the statistical strength and external applicability of the findings. The inhibition zones observed in the Kirby–Bauer assay indicate the ability of the tested samples to prevent bacterial growth; however, this method does not differentiate between bacteriostatic and bactericidal effects. To determine whether true bacterial killing occurs, further quantitative assays such as CFU enumeration or time-kill studies are required. In addition, this study did not investigate the molecular or cellular mechanisms underlying the observed antibacterial activity, and therefore the proposed biological explanations remain hypothetical. Future research should explore the antibacterial mechanisms of nanofat and microfat, identify the responsible bioactive components, and optimize membrane formulations for therapeutic use in infection control. For bacterial strains with lower sensitivity to nanofat membranes, further studies should also evaluate their potential as scaffolds for controlled antibiotic release to enhance efficacy against resistant pathogens.

## Conclusions

Nanofat membranes demonstrated significant antibacterial activity against a range of clinical pathogens, including *P. aeruginosa, K. pneumoniae, S. aureus, E. coli*, and *E. faecalis*. The study found that *P. aeruginosa* exhibited higher susceptibility to nanofat membranes, though overall antibacterial efficacy did not significantly vary between compositions. This suggests that specific nanofat components may target certain bacterial mechanisms. Further research is needed to identify these active components and optimize their therapeutic potential.

## Ethical approval

Not required.

## Animal and human rights

All treatments were performed in adherence with the Declaration of Helsinki and in accordance with the standards of good clinical care following local guidelines and regulations. This article does not contain any studies with animals performed by any of the authors.

## Informed consent

All patients included in this study provided written informed consent for accessing their patients charts and extracting their data for this study. No charts were accessed if patients declined their participation in this study.

## Funding

None of the authors received any funding or financial support for the content of this article.

## Declaration of competing interest

The author, N.F-G, is a consultant for Tulip Medical™ (Tulip Medical Products, San Diego, California, USA).
